# Experimental and Modeled Assessment of Interventions to Reduce *PM*_2.5_ in a Residence during a Wildfire Event

**DOI:** 10.3390/pollutants4010003

**Published:** 2024-01-28

**Authors:** Chrissi Antonopoulos, H. E. Dillon, Elliott Gall

**Affiliations:** 1 Maseeh College of Engineering and Computer Science, Portland State University, Portland, OR 97201, USA;; 2 Pacific Northwest National Laboratory, 902 Battelle Blvd., Richland, WA 99352, USA; 3 Mechanical Engineering, University of Washington, Tacoma, WA 98402, USA

**Keywords:** PM, air quality, portable air cleaner, HVAC, air cleaning, wildfire, smoke

## Abstract

Increasingly large and frequent wildfires affect air quality even indoors by emitting and dispersing fine/ultrafine particulate matter known to pose health risks to residents. With this health threat, we are working to help the building science community develop simplified tools that may be used to estimate impacts to large numbers of homes based on high-level housing characteristics. In addition to reviewing literature sources, we performed an experiment to evaluate interventions to mitigate degraded indoor air quality. We instrumented one residence for one week during an extreme wildfire event in the Pacific Northwest. Outdoor ambient concentrations of *PM*_2.5_ reached historic levels, sustained at over 200 μg/m^3^ for multiple days. Outdoor and indoor *PM*_2.5_ were monitored, and data regarding building characteristics, infiltration, and mechanical system operation were gathered to be consistent with the type of information commonly known for residential energy models. Two conditions were studied: a high-capture minimum efficiency rated value (MERV 13) filter integrated into a central forced air (CFA) system, and a CFA with MERV 13 filtration operating with a portable air cleaner (PAC). With intermittent CFA operation and no PAC, indoor corrected concentrations of *PM*_2.5_ reached 280 μg/m^3^, and indoor/outdoor (I/O) ratios reached a mean of 0.55. The measured I/O ratio was reduced to a mean of 0.22 when both intermittent CFA and the PAC were in operation. Data gathered from the test home were used in a modeling exercise to assess expected I/O ratios from both interventions. The mean modeled I/O ratio for the CFA with an MERV 13 filter was 0.48, and 0.28 when the PAC was added. The model overpredicted the MERV 13 performance and underpredicted the CFA with an MERV 13 filter plus a PAC, though both conditions were predicted within 0.15 standard deviation. The results illustrate the ways that models can be used to estimate indoor *PM*_2.5_ concentrations in residences during extreme wildfire smoke events.

## 1. Introduction

Wildfire frequency throughout the world continues to increase. Climate change is a major culprit, increasing the potential for wildfires, especially large-scale “megafires” [1,2]. During wildfire events, combustion products are released that include fine and ultrafine particulate matter, complex gaseous compounds that include nitrogen oxides, carbon monoxide, methane, and hundreds of volatile organic compounds (VOCs) and oxygenated VOCs (OVOCs) [3]. Prior studies have found that exposures to wildfire smoke increase mortality risk [4], respiratory illness, and cardiovascular mortality [5–9]. Like other types of pollutant exposures, vulnerable populations such as pregnant women, children, and the elderly [10] have higher risks for health effects [11,12]. Some studies have also found that low-income and indigenous populations are at greater risk of adverse effects from indoor exposures to wildfire pollutants [13–15]. Burke et al. found that occupant behaviors vary widely during smoke events [16]. Considerations such as home age, location, and occupant incomes are intersectional and challenging to model for those in the building science community.

Our research team has previously worked to understand the building energy performance of homes in the Pacific Northwest using data collected by the U.S. Department of Energy’s Home Energy Score program [17]. As we work to help the building science community increase resiliency to large wildfire events, we need to understand how simplified building metrics can be used to better understand health impacts to home residents.

One study looked at potential impacts of wildfire interventions in residential buildings that included combinations of forced air system operation, filtration, and air cleaners on health, finding that interventions could decrease both hospital admissions and deaths attributed to wildfire smoke [18]. This study adopts two similar interventions that were both measured and modeled using data gathered during a large wildfire event in Portland, Oregon in 2020. This event brought record-breaking air pollution, with sustained *PM*_2.5_ measured over 200 μg/m^3^ for multiple days, and the air quality index (AQI) reaching levels higher than 500 (>500.4 μg/m^3^ for *PM*_2.5_), the highest level captured by the AQI system. Using experimental data and mass balance modeling, we ask the following research questions in the context of simplified building science models that could be scaled:

1. What are the measured *PM*_2.5_ concentrations inside a home during a large fire event, and what is the ratio of indoor/outdoor (I/O) levels?
2. How do interventions such as high-efficiency filtration and portable air cleaners impact indoor *PM*_2.5_ concentrations?
3. How do empirical measurements of I/O ratios with indoor particle removal interventions compare to those predicted by mass balance modeling?

## 2. Background

Wildfires can significantly impact regional air quality, sometimes causing increased levels of *PM*_2.5_ that greatly exceed the daily average ambient air quality standards for days at a time [19,20]. During large wildfire events, public health officials encourage residents to stay indoors, keep windows closed, and use portable air cleaners to lower the risk of smoke inhalation [21–23]. This approach has been reviewed by large data studies, including O’Dell et al. [24].

Even with these precautions, *PM*_2.5_ levels have been shown to increase significantly inside residential buildings during wildfire events [15,25–27]. Residential buildings vary dramatically in terms of building construction; a previous study has found that the level of protection against pollutant exposure during wildfires is highly variable and dependent on housing characteristics and ventilation [28]. Properly sized portable air cleaners have been shown to decrease *PM*_2.5_ concentrations in residences during wildfires [21,22,27] and the US EPA recommends creating “clean rooms” by limiting smoke entry, keeping cool, and using portable air cleaners to filter the air [29].

Prior studies have approached both energy efficiency and air quality for residential homes, external to the influence of large wildfire events. A large body of work has focused on study of indoor air quality that includes analysis of outdoor and indoor sources, health impacts, and pollutant reduction methods along with the energy performance of the building [30–37]. In this discourse, researchers perform detailed in-home measurements and develop models to better understand with precision the way that indoor air quality is impacted by occupant behavior, stack effect, building characteristics, and HVAC operation. This body of research has created a set of modeling approaches for indoor air quality that are based in part on data that would not typically be available to a homeowner or policy maker.

Studies focused on building energy efficiency often approach residential homes using a simplified single-zone model that may serve as a proxy for large policy studies [38]. For this project, our goal is to test a simplified model for indoor air quality with plans to apply it to a large database of homes with basic housing characteristics and operation data.

## 3. Methods

A residential building in Portland, Oregon, was instrumented during the Riverside, Beachie Creek, and Lionshead fire complexes that pushed large smoke plumes to the Portland metropolitan area for nearly two weeks in September 2020. Building, infiltration, and heating, ventilation, and air conditioning (HVAC) system characteristics along with measured indoor and outdoor *PM*_2.5_ data were gathered during the study period of 12–19 September 2020.

Daily averaged outdoor ambient concentrations of *PM*_2.5_ during the experimental period ranged from 34 to 465 μg/m^3^, measured by the nearest Department of Environmental Quality (DEQ) station [39]. Together, these data were used to build a mass balance model using some assumptions developed by Fisk and Chan [18].

### 3.1. Building Instrumentation and Pollutant Data Collection

#### 3.1.1. Building Characteristics

The Northeast Portland residence that we instrumented was built in 1928. It has an above-grade volume of 243 m^3^ and two stories, and includes a partially finished basement, translating to approximately 457 m^3^ of total volume (Table 1). The home is equipped with an HVAC system that includes a central forced air gas furnace and a packaged air conditioner, and has manually operated exhaust ventilation in the bathrooms and kitchen. The central gas furnace is rated as having 92 annual fuel utilization efficiency (AFUE), and the air conditioner is a 3-ton 13 seasonal energy efficiency ratio (SEER) outdoor packaged unit. There is an additional air filtration system attached to the furnace air handler with a high-efficiency capture filter (minimum efficiency rated value [MERV] 13). Windows and doors were closed throughout the measurement timeframe, and cooking was limited.

#### 3.1.2. Air Leakage, Envelope Infiltration, HVAC Operation, and Portable Air Cleaner

Air leakage in the building envelope was measured using a TEC Minneapolis Blower Door System and DG-700 digital manometer [1], in compliance with ASTM E779–10 [40]. Blower door tests are common tools used by the building science community and would be easily available to an energy expert. Blower door testing has been confirmed as an accurate tool for helping to model particulate matter [41]. The measured airflow values during depressurization and pressurization were averaged and used to calculate the air changes per hour at 50 pascals pressure differential (*ACH*_50_), which is the most common method for assessing envelope air leakage in existing residential buildings. The *ACH*_50_ value was translated to a simplified annual averaged infiltration rate using the Lawrence Berkeley National Laboratory infiltration model [42].

HVAC operation was monitored using airflow anemometers at HVAC registers throughout the house. HVAC duty/state operation was monitored as an airflow rate in m^3^/min to determine when the HVAC system was operational; airflow rates higher than 1 m^3^/min were considered in operation. Register size was measured and duct diameter was included in airflow measurements. The exhaust ventilation system was turned off, and remained off during the duration of the measurements since ventilation airflow was not able to be filtered through the central air handler [36].

A portable air cleaner (PAC) with a “real HEPA” filter (stated as described in the manufacturer’s specifications Oransi MJR01, Radford, VA USA [43]) was added to the 14 m^2^ room with the air quality measurement equipment on day four of the experimental period. The manufacturer specified a clean air delivery rate (CADR) of 398 m^3^/h for both dust and tobacco smoke [2]. We installed only one PAC centrally in the home, co-located in the room with the *PM*_2.5_ sensors.

During the analysis of the data collected, we did not attempt to capture high-resolution data that would indicate stack effects, tracer gas studies, and detailed occupant behavior. This was intentional, since the goal of our work is a coarse model that captures larger impacts of the home performance that can be generalized for many homes on a city or neighborhood scale.

#### 3.1.3. Air Quality Measurements

Measurements were taken in two primary locations: an outdoor station set up in a backyard and an indoor station set up in the dining room. The *PM*_2.5_ monitor (Clarity Node) was equipped with an optical particle sensor (laser light scattering with remote calibration). Table 2 provides an overview of air quality measurements and equipment specifications used in the test home.

All the PM values in the paper have been corrected-based manufacturer calibration data. Low-cost monitors are known to overpredict *PM*_2.5_ during wildfire smoke events, with some development of adjustment factors to correct this overprediction [44,45]. To correct the Clarity Node measurements, we used the manufacturer-developed correction, which was developed for the 2021 fire season [46]. All subsequent discussion of experimental results is based on the corrected values for PM.

### 3.2. Mass Balance Modeling

A mass balance model was developed using a combination of parameters measured at the experimental home and from the literature, similar in concept to the model presented by Fisk and Chan [18]. Our mass balance model investigated two interventions. The first intervention is the use of an intermittently operating central HVAC air handler with a high-capture filter (MERV 13), which we reference as “HVAC” in this paper. The second intervention studied was the addition of a PAC (clean air delivery rate, or CADR of 398 m^3^/h for smoke) to the living room in the studied home. Note that, during the second intervention, the HVAC intervention continued operating based on cycling of the blower. We reference this second intervention as “HVAC+PAC” in this paper. The PAC was located in the same room as the indoor air quality monitor; we note that other zones in the home likely had elevated concentrations of *PM*_2.5_ compared to the measurements made in proximity to the PAC [47]. A summary of the scenarios that we tested is shown in Table 3.

Each intervention corresponds to assumptions that align with literature values from Fisk and Chan interventions titled “i3” and “i3.5”, respectively [18]. Model parameters needed to calculate indoor *PM*_2.5_ concentrations and I/O ratios were measured in the test home, derived from manufacturer specifications, or identified in the literature (Table 4). To estimate HVAC filter efficiency, we adjusted the capture efficiency values per Fisk and Chan and Vershaw et al. [18,48], which propose that MERV 13 filtration efficiency is reduced to approximately MERV 10 due to filter bypass. The current model used a 0.30 value, a consistent value for filter efficiency specifically related to *PM*_2.5_ (Table 4) [49]. Whenever possible, we tried to base the model parameters on averages for typical residential homes based on published literature or something a homeowner could measure, or calculated from basic home information common use in simplified energy models (like HVAC flow rates).

A quasi-steady-state model was used to predict indoor concentrations of *PM*_2.5_ as a function of parameters shown in Table 4. The mass balance equation for the home was a single, well-mixed zone as shown in Equation (1):

(1) $$C_{N}=\frac{P\lambda_{V}}{\lambda_{V}+\lambda_{D}+\lambda_{HVAC}+\lambda_{PAC}}\cdot C_{o}$$

where the concentration (*C*) is determined based on the outside air concentration (*C*_*o*_), the particle penetration factor (*P*), and system efficiencies (*λ*). The source and removal processes shown in Equation (1) are determined experimentally or based on the rating of air filters as shown in Table 4. Note that, for the period of the measurements where only HVAC filtration was operating and no PAC is present, Equation (1) is solved with *λ*_*PAC*_ = 0. While approximation of the full home as a single zone is a large simplification, it is consistent with the type of energy models for most residential homes that we are trying to align our work with.

The rate of removal by the home air conditioning system (*λ*_*HVAC*_) was calculated based on the air flow rate of the forced air blower (*Q*) normalized by air volume of the home (*V*), the duty cycle (*D*), and the filter efficiency (*ϵ*). The rate of removal by the PAC (*λ*_*PAC*_) was determined from the manufacturer’s reported clean air delivery rate, noted previously to be 398 m^3^/h.

(2) $$\lambda_{HVAC}=(Q/V)\cdot D\cdot \epsilon_{H}$$

(3) $$\lambda_{PAC}=CADR/V$$

An example for the case of the HVAC only is shown below.

(4) $$K_{HVAC}=P\cdot \frac{\lambda_{V}}{\lambda_{V}+\lambda_{D}+\lambda_{HVAC}}$$

(5) $$C_{HVAC}=K_{HVAC}\cdot C_{o}$$

To address the changes in the system over time, we adopted Euler’s method [51] for calculating the model parameters for specific time steps. We first analyzed the rate of change in the system to determine the most appropriate time steps as discussed below. Once the time step was fixed, we calculated the concentration for the model for that time step. This allowed us to adjust the time variable parameters like duty cycle for each time step in the model.

### 3.3. Sensitivity and Error

To determine the performance of the model, the root mean square error (*RMSE*) and mean absolute error (*MAE*) were calculated. We used the standard formulation for each of these calculations, determined from the raw experimental data (*y*_*i*_) and the model prediction $\left({\hat{y}}_{i}\right)$.


(6) $$MAE=\frac{1}{n}\sum_{i=1}^{n}\left|y_{i}-{\hat{y}}_{i}\right|$$

(7) $$RMSE=\frac{1}{n}\sqrt{\sum_{i=1}^{n}{\left(y_{i}-{\hat{y}}_{i}\right)}^{2}}$$

To evaluate the sensitivity of the mass balance model, we conducted an analysis of each of the input variables shown in Table 4. Each variable was varied by 10% from the mean or baseline value shown. The impact of each variation on the I/O ratio calculated for the home was recorded, assuming the HVAC+PAC scenario, and is shown in Table 5.

The sensitivity analyses of the model inputs were all less than 10% (the variation introduced). The highest sensitivity of the model was for the particle penetration factor, a variable that we determined from the literature. The uncertainty for this parameter and others taken from the literature has been evaluated in prior work [18]. Therefore, although the model is most sensitive to this input parameter, the value has been well verified experimentally. In future work, a simple method for estimating the penetration factor was developed by Zhao and Stephens [52] that could be added to the experimental work if a portable particle size unit is available.

Additional parameters that introduce variability in the model are measured infiltration rate and home volume. To assess for errors during the blower door test, both pressurization and depressurization modes were measured, consistent with recommendations from Walker et al. [53]. Both parameters were experimentally verified for this home.

## 4. Results

### 4.1. Experimental Results

The blower door results included values of 2560 *CFM*_50_ pressurized and 2150 *CFM*_50_ depressurized. We derived the average of 2355 for *CFM*_50_ total. The average was divided by the measured volume of the home to calculate a final value of 9 *ACH*_50_, indicating a moderately leaky envelope, in line with older US housing stock [54]. The base leakage infiltration ratio per the LBNL model for Portland, Oregon, was determined to be 22. Correction factors derived from Sherman [42] included a height correction factor (0.8), shielding correction factor (1), and leakiness correction factor (0.7). The product of the correction factors informs N, a correlation factor used to convert blower door test data to an estimate of the annualized infiltration rate, which was determined to be 12 [36]. The final infiltration rate is *ACH*_50_/*N*, which is equal to 0.71 1/h (Table 4).

**Figure 1.**
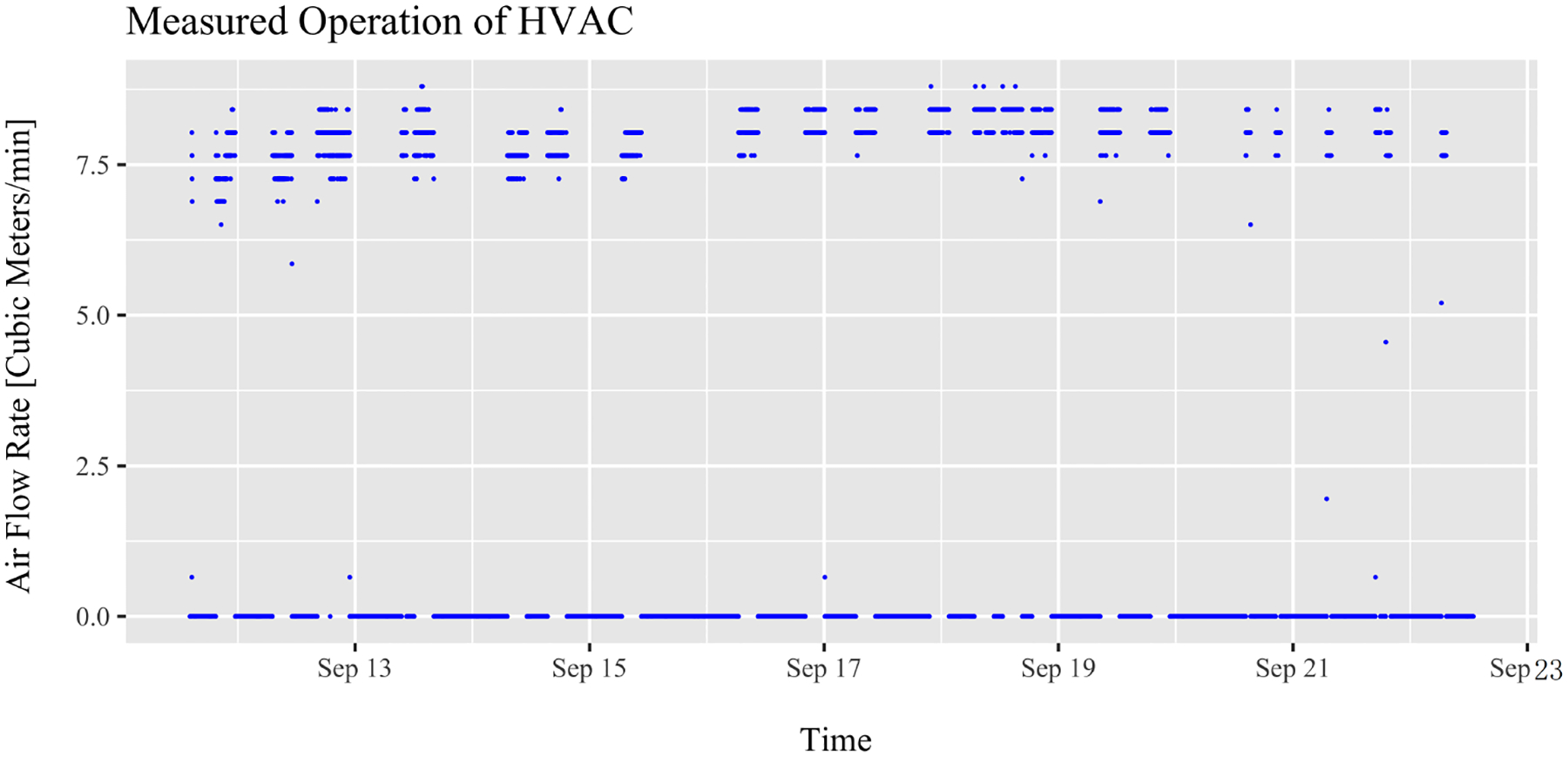
HVAC duty cycle measurements in the main living space using an anemometer. Airflow rates higher than 1 m^3^/min were considered in operation.

The duty cycle for the HVAC system was calculated from the velocity time measurements at an air supply duct in the living room using an anemometer. Flow rates higher than 1 m^3^/min were considered in operation, as shown in Figure 1. These values were used to calculate the duty cycle for each time step in the model.

**Figure 2.**
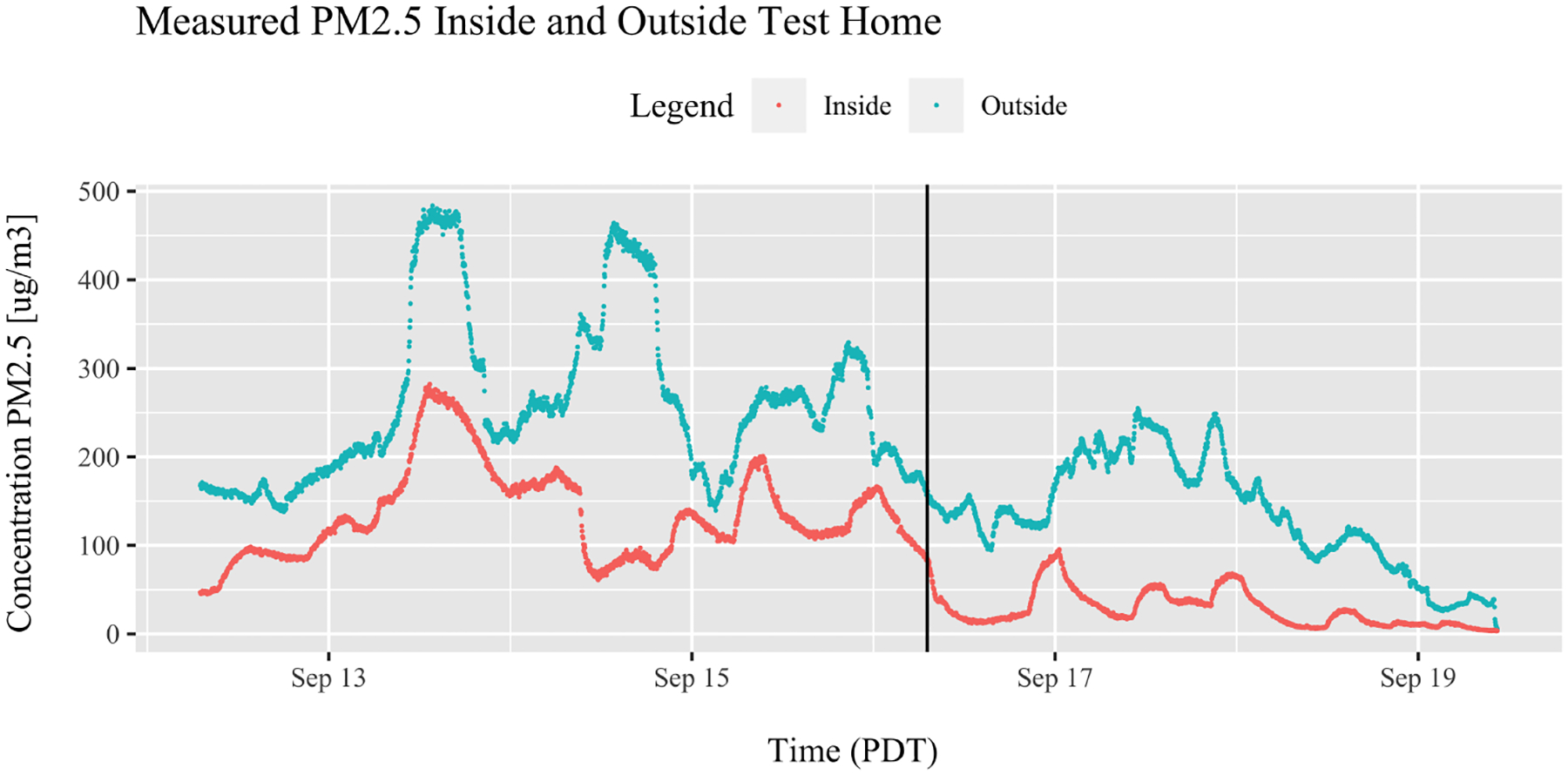
*PM*_2.5_ concentrations measured with the Clarity Node outdoors (blue) and indoors (red). The HVAC system was on for the duration, operating intermittently as shown in Figure 1. Black vertical line indicates the time when the PAC was turned on. Corrected *PM*_2.5_ concentrations are presented in Table 6.

The outdoor and indoor *PM*_2.5_ concentrations were measured using a Clarity Node monitor from 12 to 19 September 2020. All the PM values in the paper have been corrected-based manufacturer calibration data. Concentrations throughout the study period were extremely high, with peak corrected outdoor concentrations during this period reaching 484 μg/m^3^ on 13 September, with a mean value of 205 μg/m^3^ and a median value of 191 μg/m^3^ for the entire duration of the data collection period (Table 6). Measured concentrations were compared to the closest Oregon DEQ monitoring station, which at the time of this study was the SE Portland Lafayette Station, 5.8 km from the test house. DEQ monitors report 1 h averaged resolution data at the most granular level, which peaked between 528 and 542.5 μg/m^3^ on 12 and 13 September [39], compared to the Clarity Node 2 min resolution (Figure 2 and Table 3). The outdoor concentrations were highest during the earliest part of the week, before the PAC was turned on.

The maximum corrected indoor concentration reached 282 μg/m^3^, also on 13 September, with a mean value of 88 μg/m^3^ and a median value of 84 μg/m^3^ during the study period. In general, the indoor concentrations were much lower than the outdoor during the timeframe studied but followed a similar trend to the outside air (Figure 2), and the addition of the PAC significantly lowered the I/O ratio from a mean of 0.55 to a mean of 0.21 (Table 6) when it was running, which is similar to the findings from Liang et al. [26].

**Figure 3.**
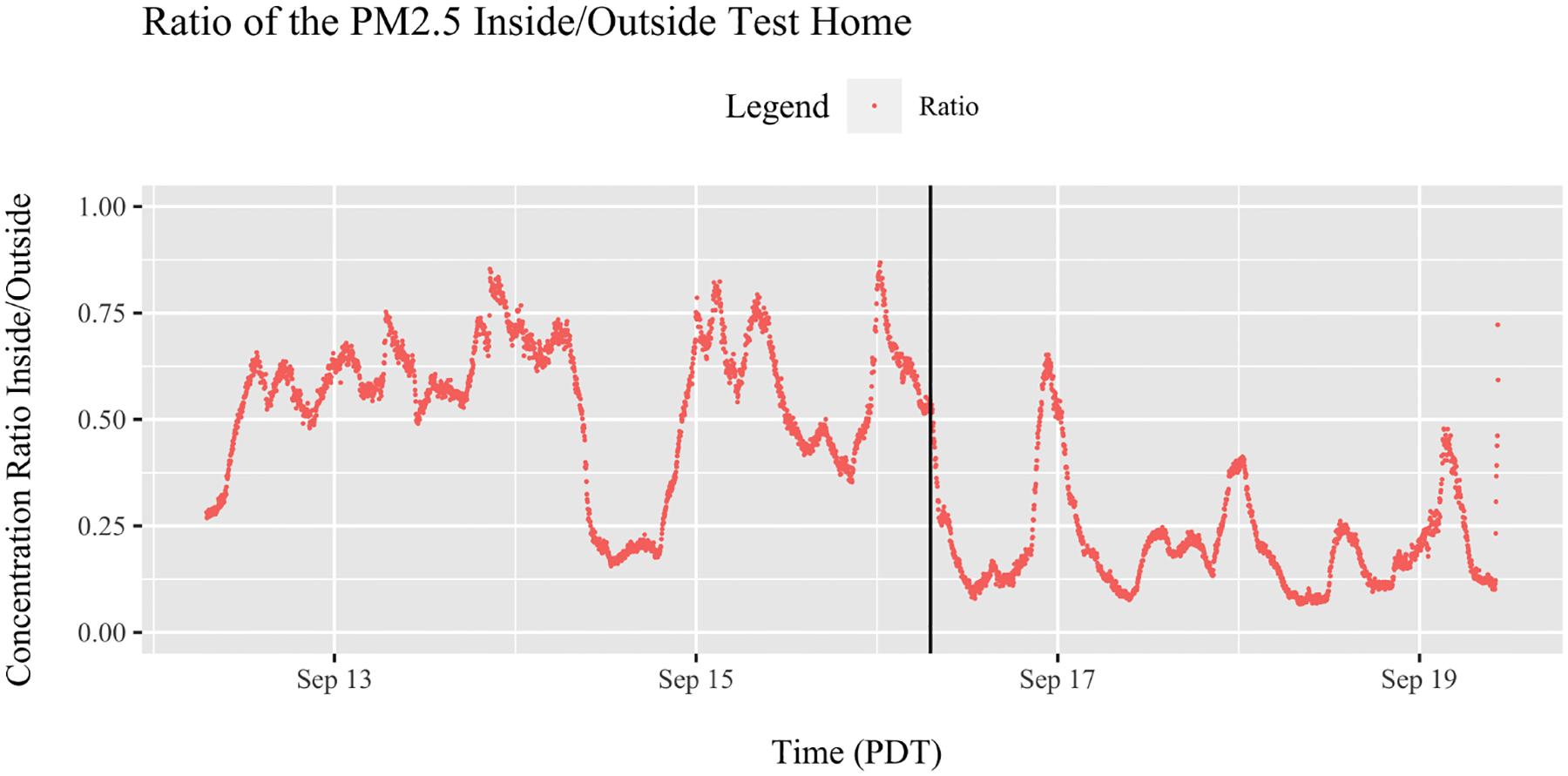
Ratio of outdoor and indoor concentrations of *PM*_2.5_ measured with the Clarity Node monitor. Black vertical line indicates the time when the PAC was turned on.

Figure 3 presents the measured indoor/outdoor ratio (I/O) of *PM*_2.5_ concentrations over time during the study period. Outdoors, there were three large peaks of *PM*_2.5_ concentrations, which caused the I/O ratio to exceed 0.75 three times, indicating significant infiltration of *PM*_2.5_. Occupants were asked to keep a journal of activities during the study period. On 13 September, occupants left the home for the entire day, leaving the house completely closed. The I/O ratios fell after the PAC was turned on, but peaked twice over 0.50. The black vertical line indicates when the PAC was turned on. Mean measured I/O ratios were 0.55 when the PAC was off and 0.21 after the PAC was turned on (Table 6).

### 4.2. Quasi-Steady-State Time Increments

The model that we developed assumes steady-state conditions for the control volume (home). This was carried out intentionally since our goal is to align our work with a larger dataset for steady-state energy operations. Because the experimental data were collected over a long period of time with variable outside air concentrations, we performed an analysis of the experimental data to determine the quasi-steady-state time increments (time steps) for further modeling using the Euler method for an approximation of a differential equation. This allowed us to compare the performance of the model with several data points over the study period.

**Figure 4.**
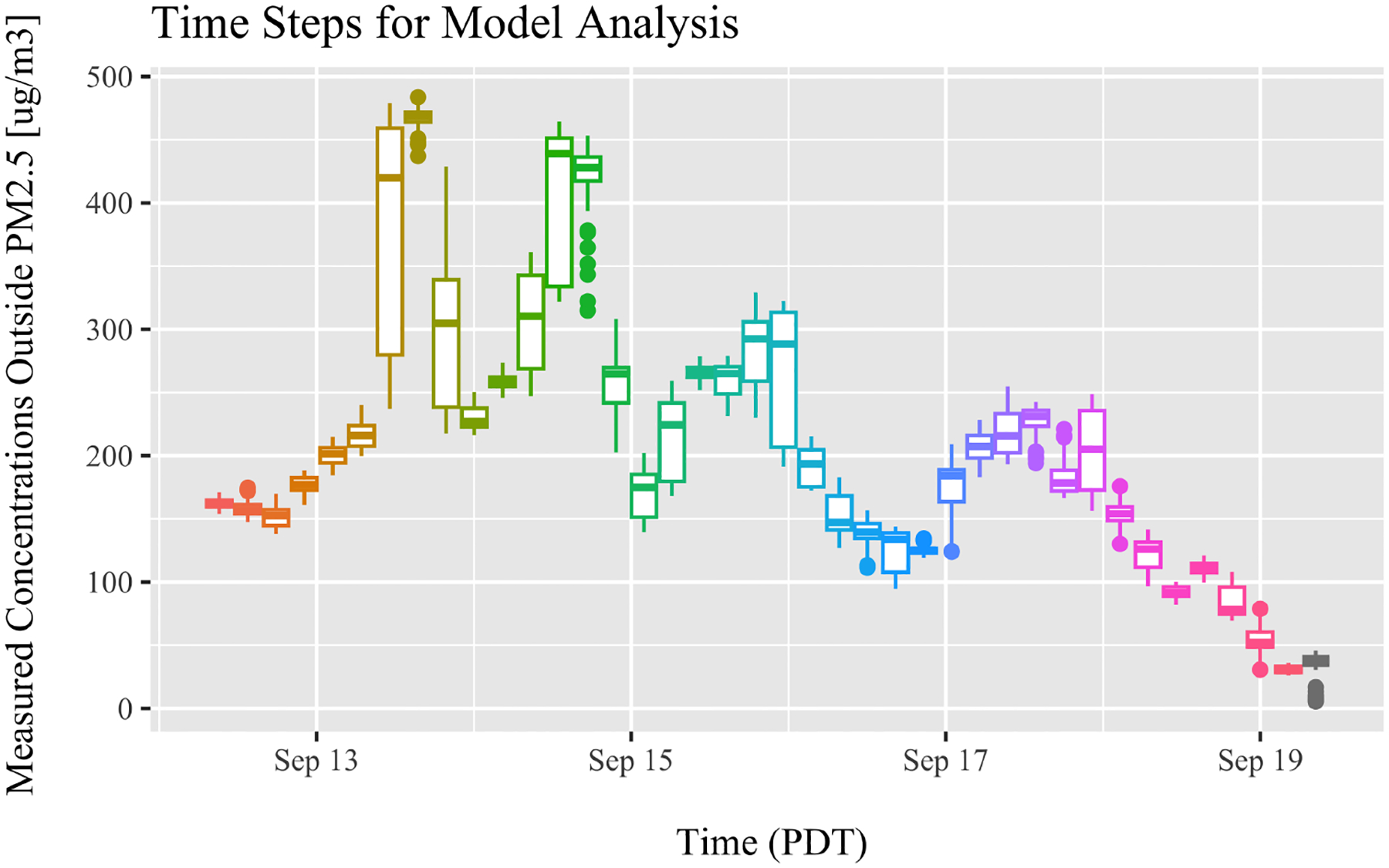
Outdoor concentrations of *PM*_2.5_ measured with the Clarity Node monitor. Each box and whisker represent a time step of approximately 4 h. The small vertical size of most of the boxes confirms that quasi-steady-state conditions were present for each time step.

We tested different time increment sizes (2–24 h), each time calculating the mean and standard deviation of the outside air concentration. We determined that 4 h time increments gave a reasonably approximation of the steady state, with variation within each 4 h time increment generally less than 2.5% from the mean of each increment. To confirm this result visually, a box and whisker plot of the outside concentration is shown in Figure 4. For each time step, the median is shown as a horizontal line inside the box. The size of the box is bounded by the upper and lower quartile. Visually, this allows for confirmation that the 4 h time increments are close to the steady state. A few of the largest boxes represent time ranges of rapid transitions in the outside air concentrations (Figure 4).

The quasi-steady-state time steps were then treated as data points for the remainder of the calculations. Other experimentally measured values, like the duty cycle, were calculated for each time step to capture the changes in the HVAC system behavior. Due to the relatively short time increments, a few time ranges had duty cycles of zero, where the HVAC system did not run.

While prior authors have used short time increments or real-time models [55], the time increments that we selected were based on our criteria of being less than 2.5% from the mean of each time increment. Lower time steps resulted in less accuracy since the HVAC system operation did not change.

### 4.3. Modeling Results

We used the mass balance model to estimate the I/O ratio for the HVAC and HVAC+PAC operating conditions. Because the mass balance model is based on steady-state operation, only one I/O ratio was calculated for each time step (Table 7). The model estimated the I/O ratio within one standard deviation across both the HVAC and HVAC+PAC interventions, as shown in Table 7.

**Figure 5.**
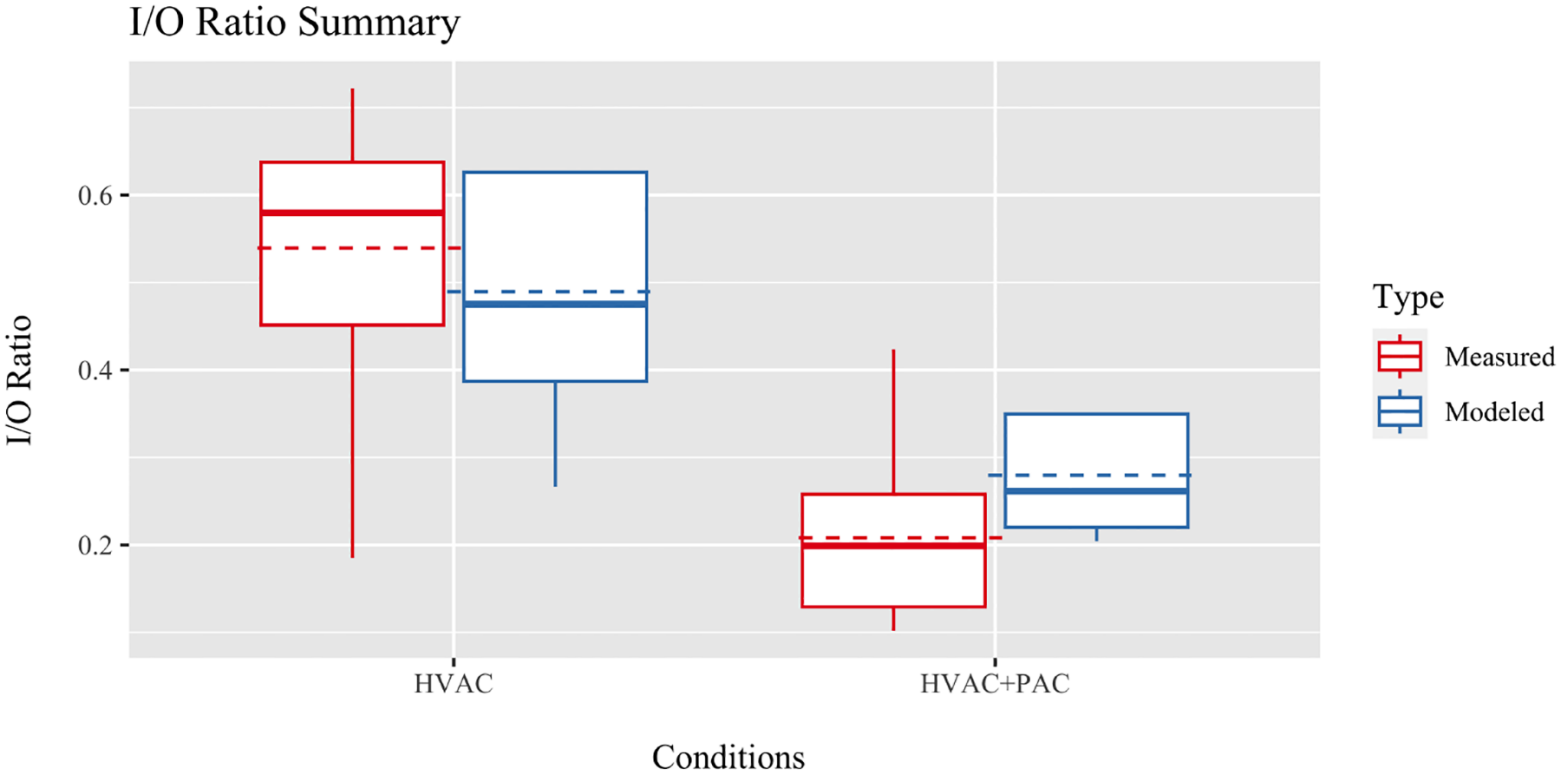
Measured I/O ratios (red/left) with the modeled I/O ratios (blue/right) for each time steps (4 h). Boxes show interquartile ranges with mean values denoted by the dotted horizontal lines, median values denoted by the solid horizontal lines, and outliers identified as the tails.

Measured and modeled I/O ranges over the experimental period were compared using boxplots for each condition; measured in red and modeled in blue (Figure 5). For the HVAC intervention, the mean modeled I/O ratio was lower than the measured mean, with an SD of 0.13. For the HVAC+PAC intervention, the mean modeled I/O ratio was larger than the measured mean, with an SD of 0.06.

In general, the model performed better for the HVAC+PAC intervention, due in part to the smaller variations in outside concentrations during this time period, but still underpredicted the measured performance of the PAC. We suspect that this underprediction is due, in part, to the imperfect mixing in the home; while the HVAC system recirculates air throughout the space, the indoor air monitors are placed in the same room as the PAC while the model assumes instantaneous mixing of air exiting the PAC through the entire home. The variation shown for the HVAC intervention (Figure 5) is due in part to larger variation in outside air conditions and the HVAC operation during this time. The model overpredicts the benefits of a high-efficiency air filter during daily operation. The same results are true if the time steps are longer (8 h). It is also possible that HVAC operation may alter indoor–outdoor airflows, as duct leakage and temperature and pressure differences may change infiltration rates; measurement of these phenomena is outside of the scope of this study.

In both cases, the model performance illustrates the challenges associated with modeling residential homes in a steady-state manner. Rapid changes in outdoor air quality, external temperatures that govern the operation of the HVAC system, building air exchange, indoor sources, and the behavior of the occupants all contribute to *PM*_2.5_ decay rates [26].While these sources of error may be concerning for a detailed indoor air quality model, the goal of using this model for a large dataset of homes as a course modeling tool makes this issue much less important. When considering hundreds of homes, it is not reasonable to provide a full transient analysis without some experimental data.

### 4.4. Model Performance

For each operation condition, the model RMSE and MAE were calculated to quantify the model performance. RMSE and MAE values are shown in Table 8 based on the calculations in Equations (9) and (10). For both RMSE and MAE, the lower the value, the better the model fit in general, but each is relative to the number of data points and the units of measure. For both RMSE and MAE, the model performance is better for the HVAC+PAC experiments.

We compared the magnitude of our RMSE and MAE values to experimental work that found RMSE ranges of 17–41 and MAE of 13–33 μg/m^3^ when comparing two types of sensors. For this reason, the slightly higher variation found for a model seems reasonable, since we would not expect it to perform as well as two redundant sensors. Furthermore, the standard deviations (SDs) of each modeled mean values were low; the HVAC intervention had an SD of 0.13 and the HVAC+PAC intervention had an SD of 0.06, indicating that modeled results for both interventions are likely to be reasonable estimations of true indoor conditions during large outdoor wildfire smoke events.

## 5. Discussion

The goal of this study was to collect empirical data of indoor and outdoor *PM*_2.5_ concentrations in a home experiencing elevated outdoor air pollution during a major wildfire, and to perform an experimental assessment of an indoor air mass balance model using those data. The mass balance model was designed to represent a simplified one-zone residential home that could be scaled to represent hundreds of homes in the Pacific Northwest with variations in size and building characteristics.

Understanding expected building performance and occupant exposure risk during smoke events is an important public health issue, particularly as wildfire events become more prolific and exacerbated by climate change [56]. Occupants of older, underperforming homes with leaky enclosures are at particular risk for degraded IAQ from wildfire smoke. Simplified residential models of this kind may be adapted to understand the policy and energy interventions most appropriate at a city scale, similar to approaches that identify retrofit opportunities for energy efficiency.

Concentrations throughout the study period were extremely high, with peak measured outdoor concentrations during this period reaching a high of 484 μg/m^3^ on 13 September, with a mean value of 205 μg/m^3^ and a median value of 191 μg/m^3^. Indoor concentrations reached 292 μg/m^3^, with a mean concentration of 88 μg/m^3^. The I/O ratio of *PM*_2.5_ peaked over 0.75 three times during the study period. When the PAC was added, I/O ratios dropped considerably, supporting the use of air-cleaning devices during wildfires. The mass balance model assumptions about the performance of a central HVAC with high-capture MERV 13 filtration running intermittently overpredicted the benefit of the upgraded high-MERV filter in the HVAC system (I/O ratio 0.55 measured, 0.48 modeled), which is similar to other studies [27]. It may be appropriate to further examine the assumptions for filtration performance in heavy smoke conditions like those observed during the experimental work. However, the SD of 0.13 indicates that the modeled values are within a reasonable error.

The benefits of a portable air cleaner with a high-efficiency HVAC (HVAC+PAC) were more closely predicted during the measured event (I/O ratio 0.22 measured and 0.28 modeled). The performance of the PAC during a heavy smoke event resulted in reduced concentrations. However, the smoke concentrations inside the home are still very high and represent a large public health risk associated with increased prevalence of wildfire events that push large plumes of smoke into densely populated areas. Only about 50% of homes in Portland, Oregon have central air conditioning systems, which means that a significant number of homes are not able to rely on a central forced air system for filtration. This also means that a substantial number of occupants are likely to open windows at night to cool their home or use window, or other portable air conditioners that rely on unfiltered outdoor air. Increasing educational campaigns to encourage residents to run central forced air systems in “fan-only” mode during wildfires could be a beneficial public safety campaign. Campaigns that distribute portable air cleaners and/or educate the public about these devices are also important, especially given the number of homes without central air systems. When the cost of a PAC is considered, it is likely that many lower-income households would be at much higher risk. Equity is an important consideration, as low-income and vulnerable populations have higher risks associated with pollutant exposure and access to air conditioning in homes [15,57,58]. High-capture filtration and potable air cleaning can be out of reach for many households, leaving them at higher risk for indoor pollutant exposure during large smoke events. The general efficiency of the building structure is important too, such as weatherization measures to limit pollutant infiltration through cracks and openings in the building envelope.

## 6. Conclusions

Interventions to improve IAQ in homes are becoming more important as wildfires increase significant smoke events, especially in the Western United States. This study categorized residential building characteristics, measured indoor and outdoor *PM*_2.5_ concentrations during a large wildfire, and evaluated the impacts of an upgraded MERV 13 filter in the CFA system and the upgraded MERV 13 filter in combination with a PAC. The results demonstrate that the combination of the high-MERV filter and the PAC reduced the indoor concentrations of *PM*_2.5_ more than the MERV 13 filter alone, but concentrations were still above the 24 h EPA exposure threshold. Future work may include additional statistical models to further explore the behavior.

The simplified single-zone mass balance model predicted indoor *PM*_2.5_ concentrations for both interventions within one standard deviation, even when outdoor concentrations were highly variable. This confirms that simplified models based on limited knowledge of building characteristics may be used to gauge relative health risks for cohorts of homes of similar vintage and characteristics. The results suggest that PACs are effective for reducing *PM*_2.5_ concentrations during wildfire, but more robust approaches are needed, especially for low-income households.

## Figures and Tables

**Table 1.** Building characteristics of experimental home.

Characteristic	Value
Year Built	1928
Home Size (m^3^)	243
Volume (m^3^)	457
Attached Garage	No
Stories	2
Number of Occupants: Pets	3:2
Blower Door Results (*CFM*_50_:*ACH*_50_)	2355:9

**Table 2.** Measured air quality parameters. Device accuracy is based on manufacturer-reported specifications.

Measurement Device	Parameters	Accuracy	Resolution	Sampling Locations
Onset HOBO UX100–011 Onset HOBO U23 Pro v2	T,RH	±0.21 °C from 0 to 50 °C ±2.5% from 10% to 90%; up to ±3.5% at 25 °C including hysteresis	1 min	Indoor: central Outdoor
Clarity Node	NO_2_, CO_2_, *PM*_2.5_	0–450 μg/m^3^ for *PM*_2.5_	2 min	Indoor: central; Outdoor: backyard
Digi-Sense Vane Anemometer WD-20250	HVAC airflows	Air velocity: ±(3% + 0.2 m/s);		Indoor: living and dining room registers

**Table 3.** Summary of the intervention conditions used in the mass balance model.

	HVAC	HVAC+PAC
Central forced air system operation	Intermittent	Intermittent
Efficiency of filter in central forced air system	Upgraded to High (MERV 13)	Upgraded to High (MERV 13)
Continuously operating portable air cleaner?	No	Yes
Experiment timeframe	9/12–9/16	9/16–9/18

**Table 4.** Summary of the intervention conditions used in the mass balance model.

Parameter	Units	Values	Description
*λ*_*V*_	1/h	0.71*	Estimated annual infiltration rate. Calculated from blower door *ACH*_50_ value
*λ*_*D*_	1/h	0.39 [18]	Rate of particle removal by deposition on surfaces
*P*	-	0.82 [50]	Particle penetration factor
*Q*/*V*	1/h	4.96**	Recirculation air flow rate of the HVAC normalized by volume
*D*	-	0.28 average *	Duty cycle. Experiment time series calculation discussed in Section 4.1
*ε*_*H*_	-	0.30	HVAC filter efficiency for *PM*_2.5_. Determined from MERV rating using published methods [48,49]
*λ*_*PAC*_	1/h	0.87**	PAC filter efficiency for *PM*_2.5_ multiplied by the air flow rate of the portable air cleaner normalized by volume. Determined from manufacturer CADR specifications for portable air cleaner
*V*	m^3^	456*	Volume of the house
*Co*	μg/m^3^	Experiment time series measurement as shown in Section 4.1.	Outside particle concentration

* Measured from test home;

** manufacture- reported equipment; specifications.

**Table 5.** Results of the sensitivity analysis for the mass balance model.

Parameter	Units	Max Sensitivity [% I/O Ratio Variation]
*λ*_*V*_	1/h	6.75–7.17
*λ*_*D*_	1/h	1.65–1.70
*P*	-	3.1–10
*Q*/*V*	1/h	2.9–3.1
*D*	-	2.9–3.1
*ϵ*_*H*_	-	2.9–3.1
*λ*_*PAC*_	1/h	2.25–2.35
*V*	m^3^	5.04–5.54

**Table 6.** Outdoor, indoor, and I/O ratios of *PM*_2.5_ during the experiment. Mean, median, and ranges are presented before and after the PAC was turned on.

Parameter	Without Portable Air Cleaner (HVAC) [μg/m^3^]	With Portable Air Cleaner (HVAC+PAC) [μg/m^3^]
Outdoor *PM*_2.5_ Mean	259.9	135.1
Outdoor *PM*_2.5_ Median	241.4	134.8
Outdoor *PM*_2.5_ Range (min-max)	138.0–483.7	5.4–254.9
Indoor *PM*_2.5_ Mean	134.4	30.2
Indoor *PM*_2.5_ Median	121.8	24.1
Indoor *PM*_2.5_ Range (min-max)	56.53–262.93	10.4–72.1
Mean I/O Ratio	0.55	0.21
Median I/O Ratio	0.58	0.18

**Table 7.** Results of the mass balance model predictions for interventions of interest for residential indoor *PM*_2.5_ concentrations of particles from outdoor sources. For the model, a total of 39 time step data points were calculated.

Intervention	Intermittent High-Capture Filter (MERV 13) (HVAC)	Intermittent High-Capture Filter Plus PAC (HVAC+PAC)
Mean measured indoor concentration (μg/m^3^)	134.9 (n = 2169)	28.2 (n = 1706)
Mean measured indoor/outdoor ratio	0.55 (n = 2169)	0.22 (n = 1706)
Mean indoor/outdoor ratio: modeled and standard deviation	0.48 SD 0.13 (n = 23)	0.28 SD 0.06 (n = 16)

**Table 8.** Results of the mass balance model predictions for interventions of interest for residential indoor *PM*_2.5_ concentrations of particles from outdoor sources. For the model, a total of 39 time step data points were calculated.

	HVAC	HVAC+PAC
RMSE [μg/m^3^]	50.24	17.43
MAE [μg/m^3^]	38.89	13.03

## Data Availability

The data presented in this study are available on request from the authors.

## Abbreviations

*ACH*_50_: air changes per hour at 50 pascals
AQI: air quality index
AFUE: annual fuel utilization efficiency
CADR: clean air delivery rate
CFA: central forced air
*CFM*_50_: cubic feet per minute at 50 pascals
DEQ: department of environmental quality
EPA: environmental protection agency
HVAC: heating, ventilation, and air conditioning
I/O: indoor/outdoor ratio
MAE: mean absolute error
MERV: minimum efficiency reporting value
PAC: portable air cleaner
RMSE: root mean square error
SD: standard deviation
SEER: seasonal energy efficiency ratio
VOCs: volatile organic compounds
